# Evaluation of the Efficacy Duration of Topical Therapies in Eyes with Primary Open-Angle Glaucoma

**DOI:** 10.3390/jcm11206166

**Published:** 2022-10-19

**Authors:** Michele Lanza, Angelo Leone, Gabriele Scognamiglio, Luigi Serra, Clemente Maria Iodice, Paolo Melillo, Francesca Simonelli

**Affiliations:** Dipartimento di Specialità Mediche, Chirurgiche ed Odontoiatriche, Università della Campania Luigi Vanvitelli, 80131 Napoli, Italy

**Keywords:** glaucoma, open-angle glaucoma, topical therapy, medical therapy, tachyphylaxis

## Abstract

Background: To investigate the efficacy interval of the topical therapies available for primary open-angle glaucoma (POAG) and the ocular and systemic features potentially associated. Methods: This retrospective study included 190 patients with POAG undergoing first topical therapy, throughout a follow-up of 15 years. The patients started one topical intraocular pressure (IOP)-lowering drug within single molecules such betablockers, prostaglandin or dorzolamide, or fixed combinations such as betablockers + prostaglandin, betablockers + dorzolamide, or betablockers + brimonidine. Efficacy duration was measured as the time between the start of the therapy and the change due to IOP increase or visual field worsening. For each patient, ocular and systemic features and comorbidities were analysed to detect any significant correlation with the length of effectiveness of every drug used. Results: The molecules explored showed some discrepancies in terms of mean duration of efficacy; however, no significant differences were demonstrated (*p* > 0.05). Furthermore, when evaluating the overall cohort, no systemic or ocular features correlated significantly with the effectiveness of the molecules explored. However, the same analysis carried out upon stratifying the different groups according to the IOP-lowering drops they received, demonstrated that the drug efficacy could be influenced by several ocular and systemic features. Conclusion: Data observed in this study suggest that there is no difference in using one of the medications evaluated as first choice of treatment of POAG if the patients are accurately evaluated and the most recent guidelines are adopted.

## 1. Introduction

Glaucoma is one of the leading causes of irreversible blindness worldwide [1]. Its most diffuse form, among the several recognized, is primary open-angle glaucoma (POAG) [2], which is a multifactorial optic neuropathy involving eyes with open anterior chamber angles [3]. It is characterized by a progressive optic nerve damage owing to the loss of retinal ganglion cells and their axons and by specific visual field (VF) defects [3]. POAG affects approximately 2% of adults above 70 years of age, escalating as a considerable problem for the worldwide health [4]. The most important goal of POAG treatment is not only to lower patients’ intraocular pressure (IOP) to preserve their visual function but also to conserve their quality of life [5,6,7].

Since POAG is a chronic multifactorial disease [3], it is hard to establish a gold standard treatment among topical drugs [8], laser [9], and surgery [10,11]. Indeed, the ideal therapy should be effective in lowering IOP and avoiding fluctuations throughout the 24 h day and be characterized by a long-term effect with no risk of tachyphylaxis and/or tolerance. Furthermore, it should be widely available, inexpensive, well-tolerated by patients, and easy to self-administer [12].

It is important to highlight that the progressive decrease of efficacy after repetitive administration of drugs over time, also known as tachyphylaxis, is a well-known side effect of chronic treatments [13], including glaucoma IOP-lowering drops as well [14]. Having more information about their actual efficacy duration could be very useful for physicians to help prevent IOP spikes over time, potential VF damages, and unnecessary ocular surface impairment. Moreover, it would allow for much better follow-up scheduling as well as more appropriate timing for other treatments, such as selective laser trabeculoplasty (SLT), argon laser trabeculoplasty (ALT), or surgical procedures.

Available studies investigating drug efficacy in decreasing glaucomatous eyes’ IOP generally explored only one or two medications, reported relatively short follow-ups, and had small cohorts of patients [15,16,17,18]. In addition, there are no published studies examining factors involved in the duration of efficacy of molecules used to treat glaucoma.

To our knowledge, this is the first study that aims at comparing multiple topical, first-choice, IOP-lowering medications in a large cohort of POAG patients throughout a long-term follow-up and evaluates their interval of effectiveness as well as systemic and ocular factors likely associated to the drug-related tolerance onset.

## 2. Materials and Methods

In this retrospective study, 190 right eyes of 190 POAG patients with a mean age of 61.28 ± 11.54 years were included. Our team retrospectively analyzed the patients’ medical records, including all the examinations carried out from January 2006 to December 2021 at the Ophthalmology Unit of University of Campania “Luigi Vanvitelli”.

The study was conducted in accordance with the declaration of Helsinki, and the protocol was approved by the Ethics Committee of University of Campania “Luigi Vanvitelli”.

All the patients provided their informed consent to use their data before starting each visit for both medical and research purposes.

The main characteristics of the patients included in this study are shown in Table 1.

Patients’ eligibility was decided upon the satisfaction of several inclusion and exclusion criteria (Table 2). Only patients affected by POAG who used IOP-lowering topical treatment as first-line therapy were included. Indeed, patients with other forms of glaucoma and those who previously underwent multiple topical therapies or other kind of glaucoma treatments such as SLT, ALT, or surgical procedures were excluded. Aiming to obtain an analysis with as little bias as possible, patients who underwent ocular surgery for other kinds of concomitant conditions, such as corneal, cataract, or retinal diseases, were excluded as well. In addition, patients requiring therapy changes due to intolerance, allergy, or interactions with other systemic treatments or diseases were excluded from this study.

Furthermore, patients assuming neuroprotective treatment (citicoline or other molecules) were also excluded. In addition, if a different performance on SAP testing was observed between the two eyes of the same patient, they were excluded because of a high suspect of potential compliance issues in assuming the topical therapy. With the purpose of avoiding interference in the interpretation of SAP changes, only patients who performed exams with good reliability indexes (less than 33% fixation losses or false-negative errors, or less than 15% false-positive errors) were included in the study.

The choice of switching treatment was driven by a pathological IOP rise, considered as any uncontrolled spike over 18 mmHg, and/or due a standardized automated perimetry (SAP) worsening, defined as loss of −1.5 dB in the Mean Deviation (MD) parameter observed at 6 months follow-up or a deepening and/or enlarging of an existing peripheral defect.

Patients assessed in our unit for glaucoma or glaucoma suspect regularly undergo a routine control. This includes a check of previous examinations and clinical documentation, a complete eye visit with an evaluation of best-corrected visual acuity, an anterior segment slit lamp observation, a measurement of central corneal thickness with Pentacam (OCULUS, Wetzlar, Germany) and of IOP with Goldmann applanation tonometry (GAT) (Haag-Streit, Köniz, Switzerland), a gonioscopy obtained with a 3-Mirrors lens or a G-4 Gonio lens (Volk Optica, Mentor, OH, USA), and an indirect ophthalmoscopy.

Cup–disc ratio was assessed by very well trained physicians using a slit lamp indirect ophthalmoscopy, using a 90D magnification lens.

With regard to GAT assessment, this was acquired between 9 and 11 AM on each visit. Only the measurements performed by the two chiefs of the glaucoma office who have been in charge during the time of the study have been included in the analysis, in particular, the mean of the two IOP evaluations which were always routinely carried out.

SAP was repeated every 6 months using a Humphrey Field Analyzer (HFAII, Carl Zeiss Meditec, Dublin, CA, USA) with a size III stimulus, a Swedish interactive threshold algorithm (SITA) standard, and a 30-2 pattern.

Glaucoma diagnoses and therapeutic regimes have been made according to the patients’ conditions, abiding by the most up-to-date international guidelines [5,19,20,21,22,23,24].

Timing and reasons underlying the therapy changes, as well as the switching molecule, were regularly recorded.

Systemic and ocular features of each patient, such as general diseases, allergies, ocular comorbidities, lens status (phakic, pseudophakic, or presence of cataract), angle anomalies (pigmentation or irido–corneal synechiae), central corneal thickness (CCT), and cup–disc ratio were evaluated to detect their potential influence on the necessity of changing therapy.

Medications assumed by the patients included in this study were single molecules (betablockers (BB), prostaglandin (PG), and dorzolamide (DZ)) or fixed combinations (betablockers + prostaglandin (BB + PG), betablockers + dorzolamide (BB + DZ), and betablockers + brimonidine (BB + BR)).

For every patient, the time of the efficacy of the therapy included in this study started with the beginning of the first IOP-lowering medication administration and ended in the event of (1) switching and/or adding drugs; (2) undergoing SLT or ALT; or (3) undergoing surgery.

### Statistical Analysis

Continuous features are reported as mean ± standard deviation (SD), and categorical features are reported as count (frequency). Analysis of variance (ANOVA) was performed to explore differences in the time of duration of the therapy according to the IOP-lowering drug assumed by patients. Linear regression models were fitted by using generalized estimating equations (GEE) in order to explore the relationship between the selected variables and the time of duration of the therapy. The analysis was performed on the overall cohort and on the groups of patients stratified on the basis of the IOP-lowering drug they received. The variables were selected through a stepwise forward selection approach. Statistical analyses were performed using SPSS software (IBM Corp. Armonk, New York, version 21.0).

## 3. Results

According to the results observed, even though the mean duration of efficacy showed some discrepancies among the medications explored, results did not reach statistical significance (*p* > 0.05) (Table 3).

Furthermore, when evaluating the overall cohort, no systemic or ocular features correlated significantly with the duration of efficacy of the molecules explored (Table 4). The parameters assessed were age, sex, concomitant general diseases, concomitant ocular disease, IOP-lowering drugs, refraction, central corneal thickness, irido–corneal angle anomalies, lens status, cup–disc ratio, and mean deviation (MD) of SAP.

The same analysis was run on the different groups studied, stratified on the basis of the IOP-lowering drug they received. The results are shown in the Table 5.

In particular, a longer BB therapy effect appeared to be associated with arthrosis, allergy, phakic, or cataract crystalline lens, whereas a shorter interval duration was associated with ocular comorbidities; additionally, a longer BB + BR treatment efficacy was associated with hypertension. Regarding the BB + DZ therapy, it showed a significantly longer IOP-lowering effect in patients affected by arthrosis; moreover, the DZ effect appeared delayed if chronic obstructive pulmonary disease (COPD) was present, while the BB + PG combination was prolonged by the concurrence of a cardiopathy. Eyes treated with PG in patients affected by systemic hypertension showed a longer time of efficacy (Table 4).

## 4. Discussion

Even though medical therapy, according to the latest European Glaucoma Society (EGS) guidelines [19], is not the only first line of treatment for POAG, and even though surgery has been demonstrated to be even more effective in IOP decrease, topical drugs are still the most diffuse choice among ophthalmologists [25,26].

It is well-established that IOP-lowering molecules work in different ways, acting on several mechanisms of the aqueous humour pathway and/or production and, thus, result in different IOP-lowering effects. In order to preserve a good quality of life, patients need the most tailored approach possible, and, thus, it would be useful for physicians to have evidence-based information about the actual efficacy interval of the various glaucoma medications available. This knowledge could improve the whole eyecare delivered to this category of patients and could be even more important to handle those who could have more difficulties in obtaining access to healthcare.

According to our data, there is no difference in using one of the medications evaluated (BB, PG, DZ, BB + PG, BB + DZ, or BB + BR) as first choice treatment in POAG eyes if the treatment is tailored according to the characteristics of the patients and the current guidelines.

Moreover, the information emerging from this study would help by allowing a better treatment customization according to several factors, such as compliance (considering once-a-day therapy), pre-existing inflammatory conditions (considering treatments without PG), or intolerances.

With regard to patients’ compliance, it is important to remember that adherence is one of the essential factors of an effective glaucoma management and that, generally, patients affected by this disease tend to have low adherence and low persistence rates to the therapy [27,28]. Among the features involved in determining the patient’s adherence rate, situational and environmental contingencies or following other medication regimens simultaneously have been observed [27]. As is easy to imagine, one of the key elements is communication problems between doctor and patient, which frequently produce inadvertent changes in therapeutic regimens [27,28]. Usually, glaucoma patients show a better adherence immediately before their periodical consultations which gives a false impression of good IOP management. The consequences of this attitude are the observation of SAP defect progressions, optic nerve changes, and a frank disease progression without apparent logical reasons, which, in addition, produces much higher costs for the health system [28].

Upon stratifying the patients of the basis of the different drug received, it was found that some molecule effects appear to be sensitive to different characteristics. Indeed, arthrosis and allergy are factors associated with a reduction of the BB IOP-lowering effect, whereas a concomitant corneal or retinal disease, or a pseudophakic status are linked to longer therapies. It is possible that arthrosis and allergy are associated to a shorter duration because of their potential increase of ocular surface basal irritation that could end in patients’ compliance reduction in self-administering the therapy. The presence of a concomitant ocular disease, on the other hand, could be associated with a longer duration because patients are usually followed up more strictly, and, thus, they are more motivated in properly assuming the therapy.

In addition, the other molecules investigated have been observed to be influenced by several elements (Table 4).

The difference in results of the association of ocular and systemic features with therapy duration between the overall cohort and the groups treated with different molecules could be related to the different dimensions of each group which could influence the statistical analysis.

The limits of this study are mainly with regard to its design: it is a retrospective study and this made it impossible to evaluate other effects of the medications such as their impact on patients’ quality of life or adherence to the regimen.

Even though the cohort evaluated in our study did not provide numerically homogeneous groups of participants allocated to receive the different classes of molecules, to our knowledge, it is still one of the largest groups in the literature evaluated for such a long follow-up when compared with similar papers previously published on the topic [15,16,17,18].

## 5. Conclusions

In conclusion, according to the results observed in this study, an accurate evaluation of the glaucomatous patient with POAG is crucial to selecting a proper IOP-lowering regime, in agreement with the most relevant international guidelines. A treatment selected with the awareness of the molecule interval of efficacy, indeed, would provide an adequate IOP reduction along with avoiding unexpected VF damage progressions and a concomitant reduction of quality of life.

## Figures and Tables

**Table 1:** Main ocular characteristics of the 190 patients included in the study.

	Mean ± SD	Interquartile Range (IQR)
Age (years)	61.28 ± 11.54	18
Visual acuity (LogMAR)	0.14 ± 0.52	0.3
Spherical defect (D)	−0.04 ± 1.55	1.5
Cylinder defect (D)	−0.12 ± 0.56	0.5
Spherical equivalent (D)	−0.1 ± 1.68	1.75
Central corneal thickness (microns)	551.3 ± 37.09	52
^1^ SAP MD (db) at starting therapy	−4.45 ± 2.45	3.56
^2^ IOP (mmHg) (first diagnosis)	26.5 ± 1.51	From 24 to 28
Cup/disc ratio	0.48 ± 0.26	0.4

^1^ Standardized automated perimetry (SAP); ^2^ intraocular pressure (IOP).

**Table 2:** Eligibility criteria of patients.

Inclusion Criteria	Exclusion Criteria
POAG diagnosis	Other forms of glaucoma
IOP-lowering topical drug as first therapy	Previous laser or surgical treatments
	Allergy or intolerance to the therapy or interactions with treatments for other comorbidities
	Neuroprotective treatment
	Any kind of ocular surgery occurred during the therapy

**Table 3:** Mean duration and standard deviation in months of the therapies included in the study with statistical testing of differences.

	Mean	SD	N	Sig.
PG (prostaglandin)	39.67	41.23	48	*p* > 0.05
BB (betablockers)	36.49	40.56	68	*p* > 0.05
DZ (dorzolamide)	29.07	36.01	14	*p* > 0.05
BB + PG (betablockers + prostaglandin)	39.26	44.36	34	*p* > 0.05
BB + BR (betablockers + brimonidine)	40.58	37.02	12	*p* > 0.05
BB + DZ (betablockers + dorzolamide)	43.23	37.23	14	*p* > 0.05
Total	38.00	40.30	190	0.95

**Table 4:** Regression model for time of duration of the therapy in the overall cohort. The variables were selected through a stepwise forward selection approach.

Parameter	B Coefficient	Std. Error (SE)	Sig.
(Intercept)	24.674	6.6414	0.000
Cardiopathy	7.033	3.9720	0.077
SAP MD	0.744	0.4141	0.072

Sig.: significance; prostaglandins; SAP MD: mean deviation of standardized perimetry.

**Table 5:** Regression models for the therapy duration in the sample stratified according to the type of treatment.

Therapy	Features	B Coefficient	Std. Error	Hypothesis Test
				Sig.
BB	(Intercept)	3.94	6.57	0.55
	Arthrosis	8.46	2.02	0.00
	Allergy	6.37	2.74	0.02
	Corneal diseases	−12.07	5.58	0.03
	Maculopathy	−16.86	3.45	0.00
	Diabetic retinophaty	−16.54	3.80	0.00
	Cataract	5.21	1.83	0.00
	Phakic	6.64	2.16	0.00
	(Scale)	93.27		
BB + BR	(Intercept)	−32.33	15.58	0.04
	Hypertension	14.54	4.90	0.00
	Age	0.56	0.24	0.02
	(Scale)	54.88		
BB + DZ	(Intercept)	60.64	0.39	0.00
	Arthrosis	49.63	2.11	0.00
	(Scale)	42.87		
DZ	(Intercept)	−17.13	3.38	0.00
	COPD	23.69	3.17	0.00
	(Scale)	35.90		
BB + PG	(Intercept)	57.15	1.72	0.00
	Cardiopathy	52.16	1.72	0.00
	(Scale)	251.47		
PG	(Intercept)	17.10	5.18	0.00
	Hypertension	6.33	3.10	0.00
	(Scale)	120.80		

## Data Availability

Data available on request due to restrictions eg privacy or ethical.
